# Hyperoxia toxicity in septic shock patients according to the Sepsis-3 criteria: a post hoc analysis of the HYPER2S trial

**DOI:** 10.1186/s13613-018-0435-1

**Published:** 2018-09-17

**Authors:** Julien Demiselle, Martin Wepler, Clair Hartmann, Peter Radermacher, Frédérique Schortgen, Ferhat Meziani, Mervyn Singer, Valérie Seegers, Pierre Asfar, the HYPER2S investigators

**Affiliations:** 1 Médecine Intensive et Réanimation, Médecine Hyperbare, Centre Hospitalier Universitaire, 4, Rue Larrey, 49933 Angers Cedex 9, France; 2 LUNAM Université, Université d’Angers, Angers, France; 3 Institut für Anästhesiologische Pathophysiologie und Verfahrensentwicklung, Universitätsklinikum, Helmholtzstrasse 8-1, 89081 Ulm, Germany; 4 Klinik für Anästhesiologie, Abteilung Klinische Anästhesiologie, Universitätsklinikum, Albert-Einstein-Allee 23, 89081 Ulm, Germany; 5 Service de Réanimation Adulte, Centre Hospitalier Intercommunal de Créteil, 40, Avenue de Verdun, 94010 Créteil Cedex, France; 6 Faculté de Médecine, Hôpitaux Universitaires de Strasbourg, Service de Réanimation, Nouvel Hôpital Civil, Université de Strasbourg (UNISTRA), Strasbourg, France; 7 INSERM (French National Institute of Health and Medical Research), UMR 1260, Regenerative Nanomedicine (RNM), FMTS, Strasbourg, France; 8 Bloomsbury Institute of Intensive Care Medicine, University College London, London, UK

**Keywords:** Septic shock, Sepsis-3, Hyperoxia, Hyperlactatemia, Oxygen transport, Oxygen extraction

## Abstract

**Background:**

Criteria for the Sepsis-3 definition of septic shock include vasopressor treatment to maintain a mean arterial pressure > 65 mmHg and a lactate concentration > 2 mmol/L. The impact of hyperoxia in patients with septic shock using these criteria is unknown.

**Methods:**

A post hoc analysis was performed of the HYPER2S trial assessing hyperoxia versus normoxia in septic patients requiring vasopressor therapy, in whom a plasma lactate value was available at study inclusion. Mortality was compared between patients fulfilling the Sepsis-3 septic shock criteria and patients requiring vasopressors for hypotension only (i.e., with lactate ≤ 2 mmol/L).

**Results:**

Of the 434 patients enrolled, 397 had available data for lactate at inclusion. 230 had lactate > 2 mmol/L and 167 ≤ 2 mmol/L. Among patients with lactate > 2 mmol/L, 108 and 122 were “hyperoxia”- and “normoxia”-treated, respectively. Patients with lactate > 2 mmol/L had significantly less COPD more cirrhosis and required surgery more frequently. They also had higher illness severity (SOFA 10.6 ± 2.8 vs. 9.5 ± 2.5, *p* = 0.0001), required more renal replacement therapy (RRT), and received vasopressor and mechanical ventilation for longer time. Mortality rate at day 28 was higher in the “hyperoxia”-treated patients with lactate > 2 mmol/L as compared to “normoxia”-treated patients (57.4% vs. 44.3%, *p* = 0.054), despite similar RRT requirements as well as vasopressor and mechanical ventilation-free days. A multivariate analysis showed an independent association between hyperoxia and mortality at day 28 and 90. In patients with lactate ≤ 2 mmol/L, hyperoxia had no effect on mortality nor on other outcomes.

**Conclusions:**

Our results suggest that hyperoxia may be associated with a higher mortality rate in patients with septic shock using the Sepsis-3 criteria, but not in patients with hypotension alone.

**Electronic supplementary material:**

The online version of this article (10.1186/s13613-018-0435-1) contains supplementary material, which is available to authorized users.

## Background

According to the Third International Consensus Definitions (Sepsis-3), a diagnosis of septic shock requires a need for vasopressor treatment to maintain mean arterial pressure (MAP) > 65 mmHg *and* a plasma lactate concentration > 2 mmol/L despite adequate volume resuscitation [1]. The latter takes into account the notion that circulatory shock “*represents an imbalance between oxygen supply and oxygen requirements*” typically associated with hyperlactatemia, which “*reflects abnormal cellular function*” [2]. Applying these more rigorous criteria for septic shock could affect the results of treatment comparisons in randomized controlled trials (RCT). Indeed, a recent post hoc analysis of the VASST trial comparing vasopressin and norepinephrine in septic shock [3] demonstrated that their sample size would have decreased by about half using the Sepsis-3 criteria with a higher overall mortality. Notably, day 28 mortality was significantly lower in vasopressin-treated patients with baseline lactate levels ≤ 2 mmol/L, whereas no difference was seen in those patients with lactate > 2 mmol/L [4].

Our recently published multicenter RCT of “*Hyperoxia and Hypertonic Saline in Patients with Septic Shock (HYPER2S)*” compared mechanical ventilation either with an inspiratory oxygen concentration of 100% (F_i_O_2_ 1.0; “hyperoxia”) during the first 24 h and F_i_O_2_ set to target an arterial hemoglobin oxygen saturation of 88–95% (“normoxia”) [5]. All patients enrolled had septic shock as characterized by the need of vasopressor support (norepinephrine or epinephrine ≥ 0.1 μg/kg/min) despite 20 mL/kg of crystalloid fluid resuscitation, but without threshold values for lactatemia at inclusion. The trial was stopped prematurely for safety reasons, with a higher mortality at day 28 in the “hyperoxia” group.

Hyperoxia administration in critically ill patients remains controversial. On the one hand, hyperoxia administration was thought to be crucial to compensate the imbalance between oxygen supply and demand [2, 6], and might be interesting in improving host defense against microbes (increase in phagocytosis and killing rate [7]) by the effect of the increased formation of reactive oxygen species [8–10]. On the other hand, there is growing evidence that hyperoxia may be toxic in such situation. The physiological effects of hyperoxia are multiple and are detailed in previous reviews [8, 11, 12]. A U-shaped relationship exists between oxygen arterial pressure during the first 24 h and mortality in ICU [13, 14]. More recently, studies have suggested that a restrictive administration of oxygen could be associated with lower mortality in ICU [15, 16].

Therefore, we hypothesized that in patients with septic shock and tissue hypoxia due to dysoxia, mirrored by increased arterial lactate, the effects of high oxygen concentration might be detrimental and related to increase the formation of reactive oxygen species.

Using the database of the HYPER2S-trial, we aimed at comparing the effects of hyperoxia on mortality and organ failures in patients with septic shock according to the Sepsis-3 criteria (lactate levels > 2 mmol/L).

## Methods

For all participating centers, the study design of the HYPER2S trial was approved by the ethics committee of the Angers University Hospital. Written informed consent was obtained from all patients, their next of kin, or another surrogate decision maker, as appropriate. If patients were unable to provide informed consent and the next of kin or a designated person was not available, the inclusion procedure for emergency situations was applied. Post hoc consent was obtained in these latter patients. The HYPER2S trial was registered with Clinicaltrial.gov (NCT 01722422).

### Patient Cohort HYPER2S

We performed a retrospective analysis of data prospectively recorded during the HYPER2S trial. This RCT compared, in a two-by-two factorial design, mechanical ventilation with “hyperoxia” (F_i_O_2_ 1.0) versus “normoxia” (F_i_O_2_ set to target an arterial hemoglobin oxygen saturation of 88–95%) during the first 24 h of septic shock, and hypertonic saline versus isotonic saline for fluid resuscitation during the first 72 h of septic shock [5]. Septic shock had been identified by the need for vasopressor support (norepinephrine or epinephrine ≥ 0.1 μg/kg/min) despite 20 mL/kg of crystalloid fluid resuscitation. The trial was stopped prematurely for safety reasons after enrolment of 442 patients (434 analyzable), as both mortality at day 28 (*p* = 0.12) and mortality at day 90 (*p* = 0.16) were higher in the hyperoxia-treated patients.

**Fig. 1:**
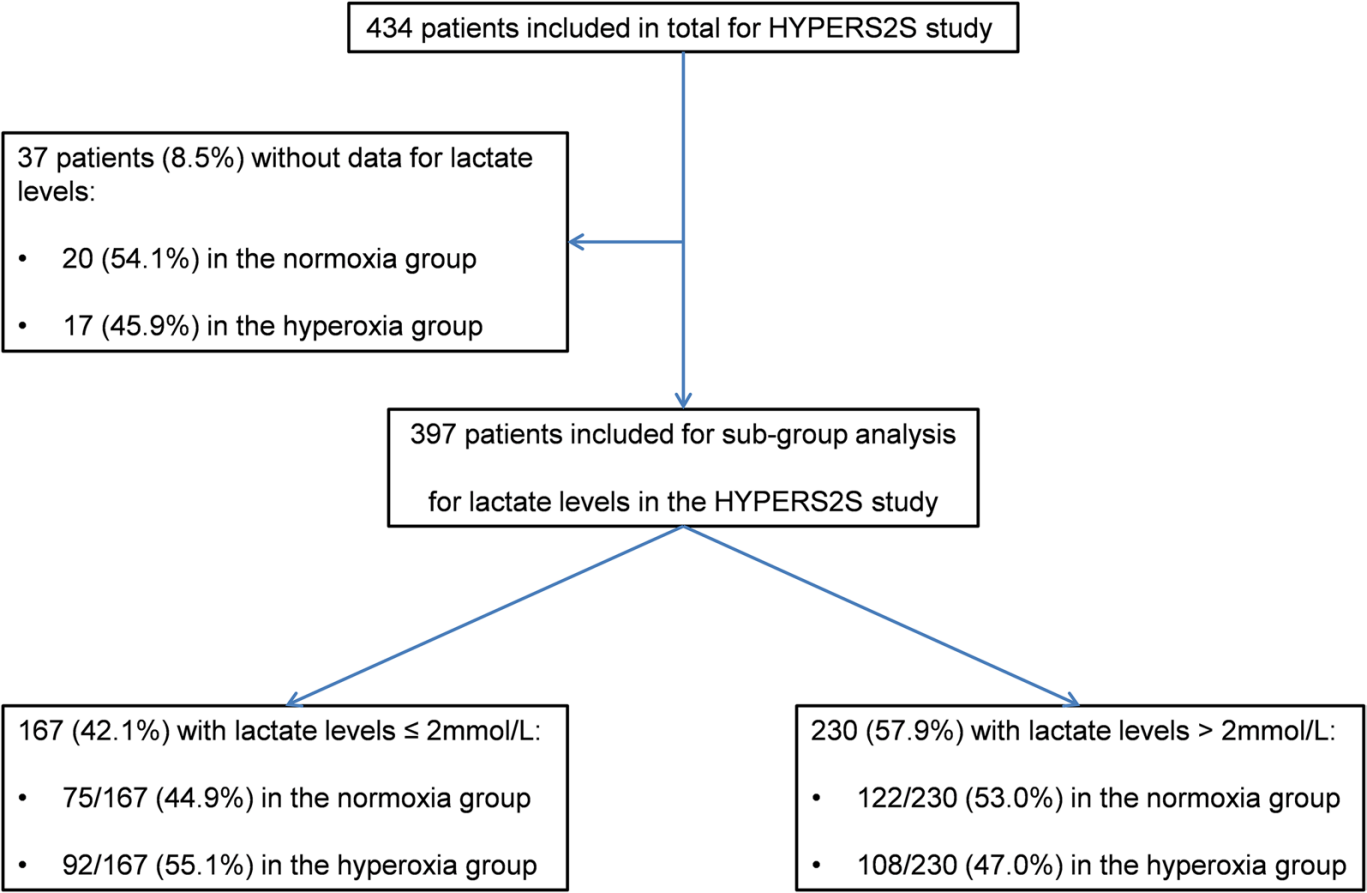
Study population

For the present post hoc analysis, we compared mortality rates in the 397 patients in whom lactate levels were available at baseline, representing 91.5% of the total study population. These patients were then subdivided into a Sepsis-3 shock subset (lactate > 2 mmol/L, *n* = 230 [53.0%]) or those with vasopressor-dependent hypotension only (lactate ≤ 2 mmol/L, *n* = 167 [38.5%]) [1] (Fig. 1). There was no significant difference in the distribution of the treatment arms between the two lactate groups (*p* = 0.110, *χ*^2^ test) (Fig. 1).

### Statistical analysis

In this post hoc analysis, only patients with available lactates at inclusion were included. Quantitative data were expressed as mean, standard deviation, median and interquartile range (IQR) for parametric and nonparametric distributions, respectively, and were compared using Student’s t tests or Wilcoxon rank-sum tests as appropriate. Qualitative variables were compared using *χ*^2^ or Fisher’s exact test. Time-to-death was illustrated with Kaplan–Meier survival curves and group comparisons were performed using the log-rank test.

To identify factors associated with survival differences from inclusion to day 28 (primary endpoint) and from inclusion to day 90 as hazard ratios (HRs), Cox regression models were computed in landmark analyses. The assumption of proportional hazards was tested by analyzing Schoenfeld residuals. In the first step, univariate analyses were conducted for every inclusion characteristics variable (including randomization arm) independently of each other. In the second step, multivariate Cox regression models were built using variables with *p* value < 0.2 in univariate analysis. When some covariates were strongly correlated (i.e., lactate and arterial pH), the most associated with survival was kept in the multivariate model. Since there was missing data for SAPS III (SAPS III was secondarily collected in the HYPER2S trial), SAPS II was chosen for the multivariate analyses. However, SAPS II and SAPS III values were well correlated (data not shown).

## Results

Patients with lactate > 2 mmol/L were more likely to have cirrhosis, but less likely to have coronary artery disease and COPD (Additional file 1: Table S1). They were also more likely to have undergone surgery, in particular emergency surgery reflecting a much higher contribution of abdominal sepsis as the source of septic shock. They were more hypotensive, albeit nonsignificant, tachycardic, more acidotic and required higher norepinephrine infusion rates (Additional file 1: Table S1). Creatinine levels and the PaO_2_/F_i_O_2_ ratio were higher (Additional file 1: Table S1). However, the number of patients with ARDS (as defined by a PaO_2_/F_i_O_2_ < 200 mmmHg) and bilirubin were similar in patients with lactate > 2 or ≤ 2 mmol/L. Accordingly, at inclusion, both SAPS II and SOFA scores were significantly higher in patients with baseline lactate > 2 mmol/L (Additional file 1: Table S1).

Throughout their ICU stay, patients with baseline lactate > 2 mmol/L needed renal replacement therapy (RRT) twice as frequently as patients with lactate ≤ 2 mmol/L and had fewer days free of vasopressor support and mechanical ventilation (Additional file 2: Table S2). Accordingly, daily SOFA scores were higher on days 1, 2, 3, and 5 (Additional file 2: Table S2). Patients with baseline lactate > 2 mmol/L had a higher mortality at day 28 (50.4% *vs.* 24.0%; *p* < 0.0001) and day 90 (55.2% vs. 30.5%; *p* < 0.0001) compared to patients with lactate ≤ 2 mmol/L (Additional file 3: Figure S1).

Except for sex ratio, no significant differences in baseline variables were seen between “hyperoxia” and “normoxia” groups for patients with lactate > 2 mmol/L (Table 1). Results were similar in patients with lactate ≤ 2 mmol/L except for a lower P_a_O_2_/F_i_O_2_ ratio in the “hyperoxia” group (Table 1).

**Table 1:** Baseline characteristics of patients according to lactate level (≤ 2 or > 2 mmol/L)

	Lactate ≤ 2 mmol/L (*n* = 167)			Lactate > 2 mmol/L (*n* = 230)				
	Normoxia (*n* = 75)	Hyperoxia (*n* = 92)	*p*	Normoxia (*n* = 122)	Hyperoxia (*n* = 108)			*p*
Age (years)								
Mean (SD)	66 (13)	66 (12)	0.877	67 (14)	69 (13)			0.360
Median (IQR)	68 (56.5–76)	67 (59.5–74.25)	0.842	68.5 (60.25–78.75)	71 (61–79.25)			0.501
Men, *n* (%)	48 (64.0%)	72 (78.3%)	0.041	80 (65.6%)	57 (52.8%)			0.048
Weight (kg)								
Mean (SD)	69.6 (16.4)	72 (17.1)	0.351	75.4 (15.5)	72.1 (16.5)			0.120
Median (IQR)	66 (59.5–74.5)	70.5 (60–80.8)	0.208	76 (65–81.8)	70 (60–80)			0.047
SAPS III								
Mean (SD)	69.6 (10)	68.9 (9.4)	0.666	74.4 (11.5)	74 (12)			0.797
Median (IQR)	68 (63.8–75.2)	70 (62.5–75)	0.921	73 (66.2–80.8)	71 (67–82)			0.614
SOFA								
Mean (SD)	9.5 (2.5)	9.6 (2.4)	0.851	10.6 (2.9)	10.6 (2.8)			0.987
Median (IQR)	9 (8–11)	9 (8–11)	0.782	11 (8–12)	11 (8–12)			0.850
Mc Cabe Score								
1	45 (60.0%)	62 (67.4%)	0.308	79 (64.8%)	70 (64.8%)			0.927
2	24 (32.0%)	20 (21.7%)		30 (24.6%)	28 (25.9%)			
3	6 (8.0%)	10 (10.9%)		13 (10.7%)	10 (9.3%)			
Recent surgical history, *n* (%)								
No	57 (76.0%)	75 (81.5%)	0.422	82 (67.2%)	67 (62.0%)			0.661
Elective	7 (9.3%)	4 (4.3%)		5(4.1%)	4 (3.7%)			
Emergency	11 (14.7%)	13 (14.1%)		35 (28.7%)	37 (34.3%)			
Preexisting disorders, *n* (%)								
Immunosuppression	14 (18.7%)	14 (15.2%)	0.553	28 (23.0%)	20 (18.5%)			0.409
Cancer	26 (34.7%)	24 (26.1%)	0.228	36 (29.8%)	35 (32.4%)			0.664
Heart failure	5 (9.3%)	7 (7.6%)	0.815	8 (6.6%)	4 (3.7%)			0.491
Chronic kidney failure	6 (8.0%)	7 (7.6%)	0.925	15 (12.4%)	12 (11.1%)			0.763
COPD	19 (25.3%)	17 (18.5%)	0.284	11 (9.1%)	16 (14.8%)			0.180
Coronary artery disease	10 (13.3%)	15 (16.3%)	0.592	12 (10.0%)	9 (8.3%)			0.678
Cirrhosis	0 (0%)	2 (2.2%)	0.502^F^	11 (9.1%)	5 (4.6%)			0.186
Source of infection, *n* (%)								
Lung	44 (58.7%)	57 (62.0%)	0.866	39 (26.2%)	37 (34.3%)			0.739
Abdomen	10 (13.3%)	15 (16.3%)		39 (32.0%)	35 (32.4%)			
Urinary tract	6 (8.0%)	6 (6.5%)		8 (6.6%)	10 (9.3%)			
Other community acquired infection	15 (20.0%)	14 (15.2%)		36 (29.5%)	26 (24.1%)			
Mean arterial pressure (mmHg)								
Mean (SD)	75 (12)	75 (12)	0.949	72 (16)	72 (16)			0.872
Median (IQR)	74 (67–80)	73.5 (67–81)	0.810	71 (64–83)	70 (61.5–81)			0.761
Heart rate (beats per min)								
Mean (SD)	96 (24)	99 (25)	0.411	108 (25)	107 (22)			0.669
Median (IQR)	94 (82–109)	96 (82–110.5)	0.578	110 (91–124)	105 (93–123)			0.688
Arterial pH								
Mean (SD)	7.33 (0.11)	7.32 (0.09)	0.766	7.25 (0.12)	7.26 (0.11)			0.222
Median (IQR)	7.33 (7.26–7.40)	7.32 (7.26–7.37)	0.716	7.27 (7.18–7.32)	7.28 (7.20–7.33)			0.177
Lactate (mmol/L)								
Mean (SD)	1.4 (0.4)	1.4 (0.4)	0.774	5.2 (3.6)	4.8 (3.8)			0.467
Median (IQR)	1.4 (1.0–1.7)	1.5 (1.0–1.7)	0.736	3.7 (2.8–6.5)	3.4 (2.7–5.2)			0.231
Crystalloid fluid treatment before inclusion (mL)								
Mean (SD)	2883 (1397)	2631 (1298)	0.234	2876 (1392)	3003 (1522)			0.511
Median (IQR)	2500 (2000–3500)	2250 (2000–3000)	0.147	2500 (2000–3500)	2500 (2000–3625)			0.684
Serum sodium (mmol/L)								
Mean (SD)	138 (5)	138 (3)	0.652	139 (6)	139 (4)			0.611
Median (IQR)	138 (134–142)	139 (136–141)	0.431	139 (136–142)	139 (136–142)			0.545
Serum chloride (mmol/L)								
Mean (SD)	107 (6)	106 (6)	0.471	106 (7)	106 (6)			0.645
Median (IQR)	106 (103–111)	105.5 (103–109)	0.448	105 (102–109)	105 (102–111)			0.355
Dose of norepinephrine (µg/kg/min)								
Mean (SD)	0.44 (0.38)	0.56 (0.65)	0.137	0.79 (0.82)	0.75 (0.74)			0.701
Median (IQR)	0.31 (0.2–0.5)	0.35 (0.2–0.65)	0.367	0.55 (0.27–1.04)	0.50 (0.30–0.88)			0.930
PaO_2_/F_i_O_2_ ratio (mmHg)								
Mean (SD)	227 (102)	191 (73)	0.012	231 (108)	232 (110)			0.934
Median (IQR)	207 (143–265)	175 (131–235)	0.023	198 (143–301)	202 (149–293)			0.876
Patients with PaO_2_ > 120 mmHg, *n* (%)	37 (49.3%)	37 (40.2%)	0.238	72 (59.0%)	61 (56.5%)			0.698
ARDS with PaO_2_/F_i_O_2_ ratio < 200 mmHg, *n* (%)	36 (48.0%)	56 (64.1%)	0.096	61 (50.0%)	52 (48.1%)			0.779

The Chi-square or Fisher test was used for qualitative data. The quantitative data were compared by t test for the mean comparison, Mann–Whitney test for median comparisons. *p* values are reported without correction of the α risk despite multiple comparisons, *p* values are presented for the comparison between “normoxia” and “hyperoxia” treatment in the lactate ≤ 2 mmol/L and lactate > 2 mmol/L groups, respectively. *F* Fisher, *IQR* interquartile range, *COPD* chronic obstructive pulmonary disease, *SAPS* simplified acute physiological score, *SOFA* sequential organ failure assessment

**Fig. 2:**
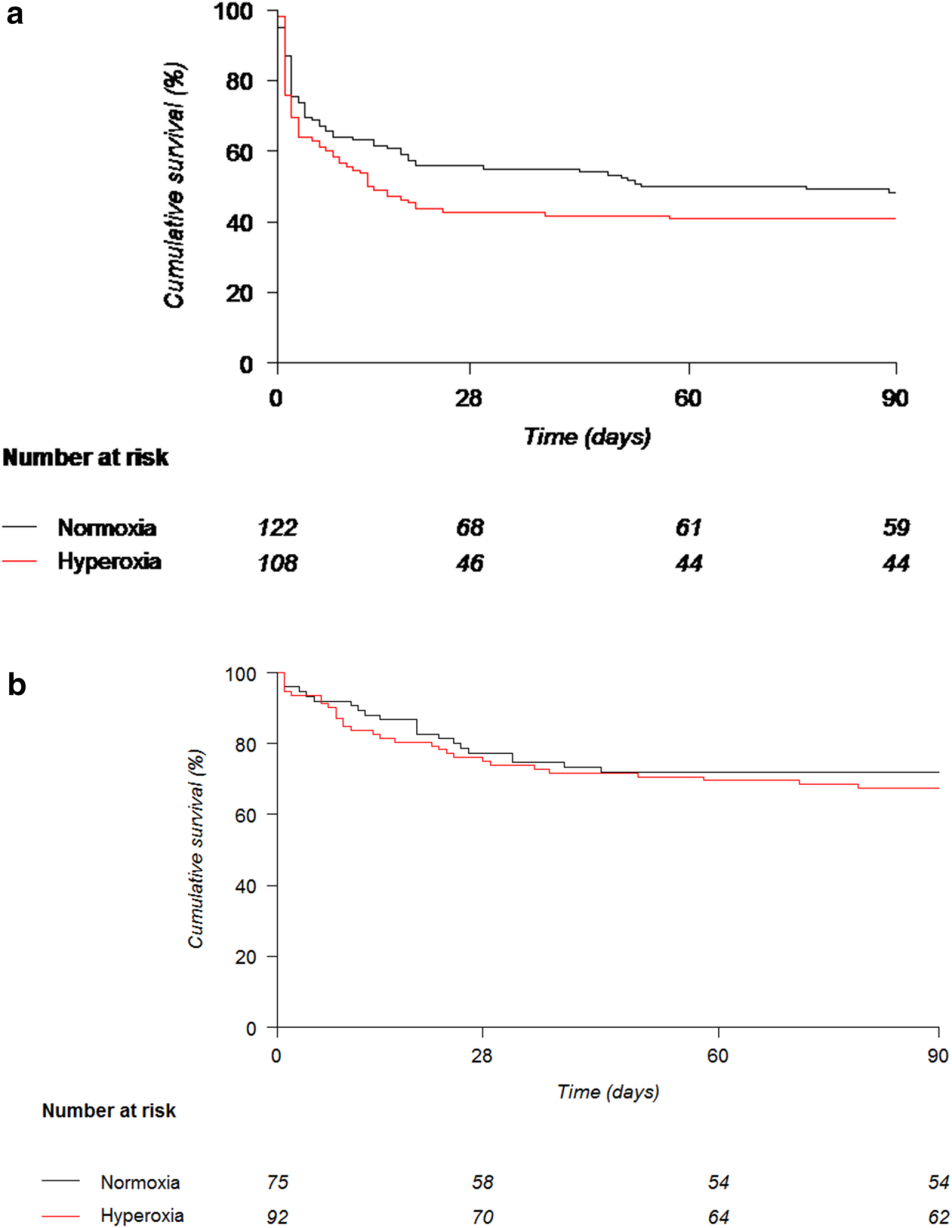
Kaplan–Meier survival curve of the “hyperoxia” (red line) and “normoxia” (black line) groups for: **a** the subgroup of patients with hyperlactatemia > 2 mmol/L at baseline. Log-rank test between the two randomization arms, *p* = 0.054 and *p* = 0.171 at day 28 and 90, respectively. **b** The subgroup of patients with lactatemia ≤ 2 mmol/L at baseline. Log-rank test between the two randomization arms, *p* = 0.680 and *p* = 0.513 at day 28 and 90, respectively

Despite a lower SOFA score at days 5 and 7 in the patients with lactate > 2 mmol/L treated with hyperoxia, mortality at day 28 tended to be higher in these patients (44.3% vs. 57.4%, *p* = 0.054) (Fig. 2a). There was no difference between groups for RRT requirements, nor for the number of RRT, vasopressor support and mechanical ventilation-free days.

In patients with baseline lactate ≤ 2 mmol/L, hyperoxia had no impact on mortality (Fig. 2b), need for RRT nor days free of RRT or vasopressor support (Table 2). However, hyperoxia was associated with fewer days free of mechanical ventilation and higher SOFA scores at days 2 and 3 (Table 2). SOFA components are detailed daily in the Additional file 4: Table S3.

**Table 2:** Evolution of the clinical characteristics and illness severity scores of patients according to lactate level (≤ 2 or > 2 mmol/L) and normoxia or hyperoxia treatment

	Lactate ≤ 2 mmol/L (*n* = 167)			Lactate > 2 mmol/L (*n* = 230)		
	Normoxia (*n* = 75)	Hyperoxia (*n* = 92)	*p* value	Normoxia (*n* = 122)	Hyperoxia (*n* =T108)	*p* value
Mortality at d28	17 (22.7%)	23 (25.0%)	0.680^¥^	54 (44.3%)	62 (57.4%)	0.054^¥^
Mortality at d90^¥^	21 (28.0%)	30 (32.6%)	0.513^¥^	63 (51.6%)	64 (59.3%)	0.171^¥^
RRT	14 (18.9%)	25 (27.8%)	0.185	50 (42.7%)	44 (41.5%)	0.853
Days without vasopressor, mean (SD)	21 (10)	19 (9)	0.323	14 (12)	12 (12)	0.149
Days without mechanical ventilation, mean (SD)	16 (9)	13 (10)	0.04	11 (11)	9 (11)	0.131
Days without RRT, mean (SD)	24 (9)	22 (10)	0.12	16 (12)	13 (12)	0.139
Survival at day 28 without organ support, *n* (%)	54 (73.0%)	61 (67.8%)	0.47	58 (49.6%)	41 (38.7%)	0.102
SOFA h0						
Mean (SD)	9.5 (2.5)	9.6 (2.4)	0.851	10.6 (2.9)	10.6 (2.8)	0.987
Median (IQR)	9 (8–11)	9 (8–11)	0.782	11 (8–12)	11 (8–12)	0.85
*n*	75	92		122	108	
SOFA h24						
Mean (SD)	9.4 (2.6)	9.8 (3)	0.273	11.9 (3.3)	11.6 (3.4)	0.526
Median (IQR)	9 (7–11)	9 (8–12)	0.335	11 (10–14)	12 (9–14)	0.693
*n*	75	89		112	104	
SOFA h48						
Mean (SD)	7.8 (3.3)	9.6 (3.6)	0.001	10.8 (4.1)	10.8 (3.6)	0.962
Median (IQR)	7 (6–10)	9 (7–12)	0.002	10 (8–14)	10 (9–14)	0.99
*n*	71	82		93	80	
SOFA h72						
Mean (SD)	6.6 (3.9)	8.4 (4.4)	0.008	9.9 (4.6)	9.2 (4.4)	0.374
Median (IQR)	6 (4–9)	8 (5–11)	0.014	10 (6.8–13)	9 (6–13)	0.43
*n*	65	82		88	71	
SOFA d4						
Mean (SD)	6.3 (4.2)	6.9 (4.4)	0.436	8 (4.7)	6.9 (3.8)	0.136
Median (IQR)	5 (3–8)	6 (4–10)	0.396	7 (4–11)	7 (4–9)	0.29
*n*	53	67		69	57	
SOFA d5						
Mean (SD)	5.9 (3.9)	6.1 (3.9)	0.815	8.3 (4.8)	6.4 (3.9)	0.024
Median (IQR)	5.5 (3–8)	5 (3–8)	0.819	7 (5–11)	6 (4–9)	0.044
*n*	44	63		62	51	
SOFA d6						
Mean (SD)	5.5 (3.8)	5.8 (3.9)	0.704	7.8 (4.8)	6.4 (4)	0.108
Median (IQR)	6 (3–7)	4 (3–9)	0.695	7 (4.5–10)	5.5 (3–9)	0.155
*n*	37	55		59	44	
SOFA d7						
Mean (SD)	5.4 (3.6)	5.8 (4.2)	0.726	8.1 (5)	4.9 (3.4)	0.001
Median (IQR)	5 (3–7)	5 (3–8)	1	7 (4–10.2)	4 (2–7)	0.002
*n*	29	47		48	34	
PaO_2_ h0						
Mean (SD)	148.4 (95.1)	126.4 (63.8)	0.09	170 (105)	151.1 (82.9)	0.139
Median (IQR)	116 (92–172)	113 (85–144)	0.289	139 (90–202)	130 (93–184)	0.295
*n*	75	92		122	108	
PaO_2_ h12						
Mean (SD)	97.1 (29.4)	265.4 (119)	< 0.0001	105.8 (44.9)	274.6 (136)	< 0.0001
Median (IQR)	89 (75–117)	273 (172–367)	< 0.0001	96 (70–124)	277 (158–364)	< 0.0001
*n*	69	86		109	103	
PaO_2_ h24						
Mean (SD)	89.3 (27.7)	221.3 (115.8)	< 0.0001	101.0 (46.2)	229.8 (131.8)	< 0.0001
Median (IQR)	82 (73–98.5)	211 (133–295)	< 0.0001	91 (77–109.2)	210.5 (119.8–322.8)	< 0.0001
*n*	67	83		100	92	
PaO_2_ h72						
Mean (SD)	88.9 (24.7)	96.5 (54.6)	0.306	88.6 (25.2)	92.4 (29.4)	0.431
Median (IQR)	84 (71–101)	82 (74–107)	0.801	84.5 (71.8–96.8)	86 (74–98)	0.481
*n*	55	69		72	61	

For mortality at day 28 and 90, respectively, an analysis in landmark with a log-rank test was used. For survival at day 28 without organ support and/or renal replacement therapy (RRT) a *χ*^2^ test was used. For the number of days without vasopressor therapy, without mechanical ventilation, and without RRT, respectively, a Student’s t test was used. ¥ = log-rank test; for mortality at day 28 in patients with a lactate > 2 mmol/L: *χ*^2^ test; *d* day, *h* hour

During the ICU stay, infectious events occurred in 69 (17.6%) patients (108 proven infectious events). There were more infectious events in the lactate ≤ 2 mmol/L group when compared to lactate > 2 mmol/L group (24% versus 12.6%, *p* = 0.003), without difference in the delay between randomization and infectious event. Nosocomial infection’s characteristics are presented in Additional file 5: Table S4.

The multivariate analysis showed that patients with lactate > 2 mmol/L treated with hyperoxia had higher risk of mortality at day 28 and 90 (HR 1.79 [1.21–2.63], *p* < 0.003, and HR 1.57 [1.09–2.28], *p* = 0.016, respectively) (Table 3). Upon the reviewing request, arguing a decrease in lactate clearance in patients with cirrhosis and septic shock, we have done the same analysis excluding cirrhotic patients. This analysis showed also a higher mortality at day 28 and 90 in patients with lactate > 2 mmol/L treated with hyperoxia (HR 1.95 [1.29–2.95], *p* < 0.002, and HR 1.69 [1.15–2.50], *p* = 0.006, respectively) (See Additional file 6: Table S5).

**Table 3:** Data analysis by using a Cox regression model with survival data censored at 28 days and then censored at 90 days in succession

	Inclusion d28				Inclusion d90			
	HR	95% CI		*p* value	HR	95%CI		*p* value
Univariate Cox model (universal analysis)								
Sex (F vs M)	1.59	1.12	2.24	0.013	1.51	1.06	2.14	0.021
Weight (per 10 kg increase)	1	0.89	1.13	0.999	1.04	0.93	1.16	0.503
pH (per 0.1 increase)	0.97	0.95	0.98	< 0.001	0.97	0.95	0.98	< 0.001
Hyperoxia vs normoxia	1.44	1.00	2.07	0.051	1.28	0.91	1.82	0.160
SAPS 2	1.05	1.03	1.06	< 0.001	1.05	1.04	1.06	< 0.001
Mac Cabe	1.25	0.97	1.62	0.089	1.25	0.98	1.61	0.073
PaO_2_/FiO_2_	1	0.99	1.00	0.860	1	0.99	1.00	0.660
Autoimmune disease	1.17	0.76	1.80	0.480	1.262	0.84	1.89	0.260
Multivariate Cox model								
Sex (F vs M)	1.55	1.06	2.27	0.022	1.53	1.06	2.22	0.024
pH (per 0.1 increase)	0.96	0.95	0.98	< 0.001	0.97	0.95	0.98	< 0.001
Hyperoxia vs normoxia	1.79	1.21	2.63	0.003	1.57	1.09	2.28	0.016
SAPS 2	1.05	1.04	1.06	< 0.001	1.05	1.04	1.06	< 0.001
Mac Cabe	1.07	0.82	1.39	0.613	1.06	0.83	1.37	0.640

*HR* hazard ratio, *CI* confidence interval, *F* female, *M* male

## Discussion

This post hoc analysis of the HYPER2S trial aimed to assess the impact of the new Sepsis-3 septic shock criteria (vasopressor-dependent hypotension *and* hyperlactatemia despite adequate fluid resuscitation [1]) on the number of patients enrolled in the study and their mortality rate, and the effect of hyperoxemia. The Sepsis-3 criteria were fulfilled in 58% of the total study population, with mortality at day 28 being more than double that of the patients with vasopressor-dependent hypotension without a raised lactate level. Hyperoxia was associated with a higher mortality rate in patients fulfilling the Sepsis-3 shock criteria, while this association was not observed in patients with vasopressor-dependent hypotension alone (Additional file 6: Table S5).

The Sepsis-3 shock criteria were derived using the Surviving Sepsis Campaign database of 18.840 unselected septic patients with organ dysfunction [17]. Patients requiring vasopressors to maintain MAP > 65 mmHg and with persisting hyperlactatemia > 2 mmol/L despite adjudged adequate fluid resuscitation had a 42.3% hospital mortality compared to 30.1% with vasopressor-dependent hypotension alone. Comparable data were seen on retrospective analysis of the VASST study database [4] and the HYPER2S study. These higher mortality rates in the Sepsis-3 shock subset were reflected by the higher illness severity scores at baseline, and the requirement for more organ support therapy.

The retrospective analysis of the VASST study [4] revealed a significant outcome benefit from vasopressin only in those patients with vasopressor-dependent hypotension alone. Mortality was identical in patients fulfilling the Sepsis-3 shock criteria.

In this present analysis, we confirm that treatment effect may vary according to the criteria used for defining septic shock. Indeed, hyperoxia had no effect on mortality albeit a longer requirement for mechanical ventilation in vasopressor-dependent hypotensive patients, whereas there was a near-significant increase in mortality (57.4% vs. 44.3% normoxia; *p* = 0.054) in the Sepsis-3 shock cohort. Hyperlactatemia despite adequate fluid resuscitation is considered to reflect more severe cellular and metabolic abnormalities, and thus places affected patients at higher mortality risk [17].

There is a growing evidence that hyperoxia may be associated with higher mortality and that conservative strategies may contribute to lower mortality [16, 17]. However, there is no certainty on the implicated pathophysiological mechanisms in oxygen toxicity. Indeed, the disparity in outcomes with hyperoxia only seen in those fulfilling the Sepsis-3 shock criteria suggests an additional toxic impact of oxygen in this more severe subset. Oxygen administration has long been considered a cornerstone in the management of patients with septic shock [9]. Circulatory shock is considered to “*represent an imbalance between oxygen supply and oxygen requirements*” [2]. While hyperoxia increases tissue oxygen tension, even in shock states with profound reduction of tissue oxygen transport [18], it can compromise macro- and microcirculatory blood flow [8, 11, 19]. After fluid resuscitation, septic shock generally has a “*distributive shock*” pattern, where the “*main deficit lies in the periphery, …with altered oxygen extraction*” [2]. In most cell types, other than erythrocytes, oxygen is crucial for sufficient adenosine triphosphate synthesis via the mitochondrial oxidative phosphorylation, acting as the final electron acceptor in the respiratory chain. Oxygen is also one of the strongest oxidizing agents capable of damaging any biological molecule due to excess production of reactive oxygen species (ROS) [20]. Although ROS can also be generated with hypoxia, ROS formation is directly related to the level of arterial and tissue oxygen tension [21]. It is tempting to speculate that under conditions of profound alterations of cellular oxygen extraction and utilization—perhaps manifest clinically as hyperlactatemia—hyperoxia, with an increase in available oxygen, may lead to excessive ROS formation with subsequent oxidative stress-induced damage. In septic shock, ROS production and damage may be amplified by impaired mitochondrial respiration and depleted antioxidant defenses [22]. Even if this study has several limitations, discussed thereafter, it could be hypothesized that hyperoxia toxicity, with an increased oxidative stress due to ROS formation, may be delayed. This mid- or long-term harmful effect of hyperoxia may explain that most variates are not significantly different between oxygenation groups, except for some major patient-centered outcome variables. Hyperlactatemia may be due to excessive peripheral production or decrease clearance such as in cirrhosis. It was suggested by a reviewer to test our hypothesis without cirrhotic patients, and these additional results support our hypothesis.

Some limitations must be underlined in this analysis. First, the HYPER2S trial was stopped prematurely for safety reason. From a strict statistical point of view, mortality difference with hyperoxia in the Sepsis-3 shock cohort was not significant (*p* = 0.054). However, this is no longer true when results are adjusted on confounders (multivariate analysis). This is likely related to lack of power, as the absolute and relative mortality at day 28 rates increased by 13.1% and 29.6%, respectively, in a sizeable number of patients (264). This suggests a clinical relevance of our results. Second, the post hoc character of the analysis, in a retrospective setting, may have missed some masked imbalance between groups and the frailty of multivariate analysis as well as multiple testing should be taken in account. Therefore, it is wise to consider these results as hypothesis generating, as the study has not the statistical power to conclude on a link between hyperoxia and mortality.

## Conclusions

Results of this post hoc analysis of the HYPER2S trial suggest that hyperoxia treatment for 24 h in patients with septic shock fulfilling the Sepsis-3 definition may be associated with a higher mortality rate. Toxic effects of oxygen were not found in patients with sepsis and without hyperlactatemia, requiring vasopressors. Our results suggest a differential effect of oxygen according to the underlying cellular and metabolic status of the patient and may vary according to SEPSIS 2 or 3 definition. Due to post hoc design of our study, our results should be considered as hypothesis generating.

## Additional files

**Additional file 1: Table S1.** Baseline characteristics of patients with lactate levels > and ≤ 2 mmol/L, respectively. For gender, recent surgical history, preexisting disorders, source of infection, and the number of patients with ARDS and with PaO_2_ > 120 mmHg at baseline a *χ*^2^ test was used. For the other parameters, a Student’s *t* test and Mann–Whitney rank sum test was used.

**Additional file 2: Table S2.** Clinical characteristics and illness severity scores of patients with lactate levels > and ≤ 2 mmol/L, respectively. For mortality at day 28 and 90, respectively, an analysis in landmark with a log-rank test was used. For survival at day 28 without any organ support and renal replacement therapy (RRT) a *χ*^2^ test was used. For the number of days without vasopressor therapy, without mechanical ventilation, and without RRT, respectively, and for the SOFA scores, a Student’s *t* test and a Mann–Whitney rank sum test was used. *SOFA* sequential organ failure assessment.

**Additional file 3: Figure S1.** Kaplan–Meier curves of *all* patients with hyperlactatemia (lactate > 2 mmol/L) (*n* = 230) and those with lactates ≤ 2 mmol/L at baseline (*n* = 167).

**Additional file 4: Table S3.** Daily evolution of the SOFA score’s components of patients according to lactate level (≤ 2 or > 2 mmol/L) and normoxia or hyperoxia treatment.

**Additional file 5: Table S4.** Infectious events during the ICU stay of patients according to lactate level (≤ 2 or > 2 mmol/L) and normoxia or hyperoxia treatment.

**Additional file 6: Table S5.** Data analysis by using a Cox regression model with survival data censored at 28 days and then censored at 90 days in succession, excluding cirrhotic patients. *HR* hazard ratio, *CI* confidence interval, *F* female, *M* male.
