# Supplementary material for: Hyperoxia toxicity in septic shock patients according to the Sepsis-3 criteria: a post hoc analysis of the HYPER2S trial
Source: Ann Intensive Care. 2018 Sep 17;8:90. doi: 10.1186/s13613-018-0435-1 (PMC6141409; doi:10.1186/s13613-018-0435-1)
Supplement: Supplementary file 1 — Additional file 1: Table S1. Baseline characteristics of patients with lactate levels > and ≤ 2 mmol/L, respectively. For gender, recent surgical history, preexisting disorders, source of infection, and the number of patients with ARDS and with PaO2 > 120 mmHg at baseline a χ2 test was used. For the other parameters, a Student’s t test and Mann–Whitney rank sum test was used. [file 13613_2018_435_MOESM1_ESM.docx]

**Additional file 1: Table S1.** Baseline chracteristics of patients with lactate levels > and ≤ 2 mmol/L, respectively. For gender, recent surgical history, pre-existing disorders, source of infection, and the number of patients with ARDS and with PaO_2_ > 120 mmHg at baseline a χ^2^ test was used. For the other parameters, a Student’s t-test and Mann Whitney rank sum test was used.

|  |  | | **Lactate ≤ 2 mmol/L**  **(n = 167)** | **Lactate > 2 mmol/L**  **(n = 230)** | **p** |
| --- | --- | --- | --- | --- | --- |
| Age (years) | Mean (SD) | | 66 (13) | 68 (14) | 0.107 |
|  | Median (IQR) | | 67 (57.5-75) | 70 (61 -79) | 0.047 |
| Men | n (%) | | 120 (71.9%) | 137 (59.6%) | 0.011 |
| Weight (kg) | Mean (SD) | | 70.9 (16.8) | 73.8 (16.0) | 0.085 |
|  | Median (IQR) | | 69 (60-78) | 74 (64.3-81) | 0.014 |
| SAPS II | Mean (SD) | | 52.4 (12.8) | 59.8 (15.8) | <0.001 |
|  | Median (IQR) | | 52 (44-58.5) | 58 (49-69) | <0.001 |
| SOFA† | Mean (SD) | | 9.5 (2.5) | 10.6 (2.8) | 0.001 |
|  | Median (IQR) | | 9 (8-11) | 11 (8-12) | 0.002 |
| Recent surgical history: N (%) | | |  |  |  |
|  | no | | 132 (79.0%) | 149 (64.8%) | 0.004 |
|  | Elective | | 11 (6.6%) | 9 (3.9%) |  |
|  | Emergency | | 24 (14.4%) | 72 (31.3%) |  |
| Prexisting disorders: N (%) | | |  |  |  |
|  | Immunosuppression | | 28 (16.8%) | 48 (20.9%) | 0.30 |
|  | Cancer | | 50 (29.9%) | 71 (31.0%) | 0.82 |
|  | Heart failure | | 12 (7.2%) | 12 (5.2%) | 0.42 |
|  | Chronic kidney failure | | 13 (7.8%) | 27 (11.8%) | 0.19 |
|  | COPD | | 36 (21.6%) | 27 (11.8%) | 0.009 |
|  | Coronary disease | | 25 (15.0%) | 21 (9.2%) | 0.075 |
|  | Cirrhosis | | 2 (1.2%) | 16 (7.0%) | 0.006 |
| Source of infection: N (%) | | |  |  |  |
|  | Lung | | 101 (60.8%) | 76 (33.0%) | <0.001 |
|  | Abdomen | | 25 (15.1%) | 74 (32.2%) |  |
|  | Urinary tract | | 12 (7.2%) | 18 (7.8%) |  |
| Other community acquired infection | | | 29 (16.9%) | 62 (27%) |  |
| Mean arterial pressure | | Mean (SD) | 75 (12) | 72 (16) | 0.067 |
| (mmHg) | | Median (IQR) | 74 (67-81) | 70 (62-82) | 0.051 |
| Heart rate | | Mean (SD) | 98 (24) | 108 (23) | 0.001 |
| (beats per minute) | | Median (IQR) | 95 (82-110) | 107.5 (92-124) | <0.001 |
| Arterial pH | | Mean (SD) | 7.32 (0.10) | 7.26 (0.11) | <0.001 |
|  | | Median (IQR) | 7.32 (7.26-7.38) | 7.27 (7.18-7.33) |  |
| Lactate | | Mean (SD) | 1.4 (0.4) | 5.0 (6.7) | - |
| (mmol/L) | | Median (IQR) | 1.4 (1.0-1.7) | 3.5 (2.7-5.6) |  |
| Crystalloid fluid  treatment before | | Mean (SD) | 2744 (1345) | 2935 (1452) | 0.18 |
| inclusion (mL) | | Median (IQR) | 2500 (2000-3000) | 2500 (2000-3500) | 0.18 |
| Creatinine (μmol/L) | | Mean (SD) | 134 (99) | 178 (118) | 0.001 |
|  | | Median (IQR) | 105 (69-165) | 151 (98-210.8) | <0.001 |
| Bilirubin (mmol/L) | | Mean (SD) | 25 (39) | 24 (31) | 0.76 |
|  | | Median (IQR) | 13 (9-22.5) | 15 (9-26) | 0.31 |
| Serum sodium | | Mean (SD) | 138 (4) | 139 (5) | 0.12 |
| (mmol/L) | | Median (IQR) | 138 (135-141) | 139 (136-142) | 0.11 |
| Serum chloride | | Mean (SD) | 106 (6) | 106 (6) | 0.52 |
| (mmol/L) | | Median (IQR) | 106 (103-110) | 105.(102-110) | 0.41 |
| Dose of noradrenaline | | Mean (SD) | 0.51 (0.55) | 0.77 (0.78) | 0.001 |
| (µg/kg/minute) | | Median (IQR) | 0.33 (0.20-0.57) | 0.50 (0.30-1.0) | <0.001 |
| PaO_2_/F_i_O_2_ ratio | | Mean (SD) | 206.9 (88.3) | 231.4 (108.9) | 0.014 |
| (mmHg) | | Median (IQR) | 187 (135-250.8) | 200.5 (147-294) | 0.047 |
| Patients with PaO_2_ >120 mmHg | | | 74 (44.3%) | 133 (57.8%) | 0.008 |
| ARDS with PaO_2_/F_i_O_2_ ratio  < 200 mmHg | | | 92 (55.1%) | 113 (49.1%) | 0.24 |
