# Supplementary material for: Hyperoxia toxicity in septic shock patients according to the Sepsis-3 criteria: a post hoc analysis of the HYPER2S trial
Source: Ann Intensive Care. 2018 Sep 17;8:90. doi: 10.1186/s13613-018-0435-1 (PMC6141409; doi:10.1186/s13613-018-0435-1)
Supplement: Supplementary file 2 — Additional file 2: Table S2. Clinical characteristics and illness severity scores of patients with lactate levels > and ≤ 2 mmol/L, respectively. For mortality at day 28 and 90, respectively, an analysis in landmark with a log-rank test was used. For survival at day 28 without any organ support and renal replacement therapy (RRT) a χ2 test was used. For the number of days without vasopressor therapy, without mechanical ventilation, and without RRT, respectively, and for the SOFA scores, a Student’s t test and a Mann–Whitney rank sum test was used. SOFA sequential organ failure assessment. [file 13613_2018_435_MOESM2_ESM.docx]

**Additional file 2: Table S2.** Clinical characteristics and illness severity scores of patients with lactate levels > and ≤ 2 mmol/L, respectively. For mortality at day 28 and 90, respectively, an analysis in landmark with a log-rank test was used. For survival at day 28 without any organ support and renal replacement therapy (RRT) a χ^2^ test was used. For the number of days without vasopressor therapy, without mechanical ventilation, and without RRT, respectively, and for the SOFA scores, a Student’s t-test and a Mann Whitney rank sum test was used. SOFA=Sequential Organ Failure Assessment.

|  |  | **Lactate ≤ 2 mmol/L (n = 167)** | **Lactate >2 mmol/L**  **(n = 230)** | **p-value** |
| --- | --- | --- | --- | --- |
| Mortality at day 28 |  | 40 (24.0%) | 116 (50.4%) | <0.001 |
| Mortality at day 90 |  | 51 (30.5%) | 127 (55.2%) | <0.001 |
| RRT * |  | 33 (20.1%) | 98 (43.9%) | <0.001 |
| Number of days without vasopressor | Mean (SD)  Median (IQR) | 20.0 (9.3)  25 (17-26) | 13.3 (11.9)  14 (0-26) | <0.001  <0.001 |
| Number of days without mechanical ventilation | Mean (SD)  Median (IQR) | 14.1 (10.3)  17.5 (2.75-23.25) | 9.9 (10.9)  1 (0-22) | 0.001  0.002 |
| Number of days without RRT * | Mean (SD)  Median (IQR) | 22.7 (9.1)  28 (22-28 | 14.7 (12.4)  16 (1-28) | <0.001  <0.001 |
| Survival on day 28 without organ support | Mean (SD) | 115 (70.1%) | 99 (44.4%) | <0.001 |
| SOFA 0h | Mean (SD) | 9.5 (2.5) | 10.6 (2.8) | 0.001 |
|  | Median (IQR) | 9 (8-11) | 11 (8-12) | 0.002 |
|  | N | 151 (90.4%) | 216 (93.9%) |  |
| SOFA 24h | Mean (SD) | 9.6 (2.8) | 11.7 (3.3) | <0.001 |
|  | Median (IQR) | 9 (8-11) | 12 (9-14) | <0.001 |
|  | N | 164 (98.2%) | 216 (93.9%) |  |
| SOFA 48h | Mean (SD) | 8.8 (3.5) | 10.8 (3.9) | <0.001 |
|  | Median (IQR) | 8 (6-11) | 10 (8-14) | <0.001 |
|  | N | 153 (91.6%) | 173 (75.2%) |  |
| SOFA 72h | Mean (SD) | 7.6 (4.3) | 9.6 (4.5) | 0.001 |
|  | Median (IQR) | 7 (4-11) | 10 (6-13) | 0.001 |
|  | N | 147 (88.0%) | 159 (69.1%) |  |
| SOFA d4 | Mean (SD) | 6.7 (4.3) | 7.5 (4.3) | 0.125 |
|  | Median (IQR) | 6 (3-9) | 7 (4-10) | 0.075 |
|  | N | 120 (71.9%) | 126 (54.8%) |  |
| SOFA d5 | Mean (SD) | 6 (3.9) | 7.4 (4.5) | 0.017 |
|  | Median (IQR) | 5 (3-8) | 7 (4-10) | 0.019 |
|  | N | 107 (64.1%) | 113 (49.1%) |  |
| SOFA d6 | Mean (SD) | 5.7 (3.9) | 7.2 (4.5) | 0.013 |
|  | Median (IQR) | 5 (3-8) | 6 (4-9.5) | 0.013 |
|  | N | 92 (55.1%) | 103 (44.8%) |  |
| SOFA d7 | Mean (SD) | 5.6 (3.9) | 6.8 (4.7) | 0.107 |
|  | Median (IQR) | 5 (3-8) | 6 (3-9) | 0.177 |
|  | N | 76 (45.5%) | 82 (35.7%) |  |
