# Supplementary figures and images for: Hyperoxia toxicity in septic shock patients according to the Sepsis-3 criteria: a post hoc analysis of the HYPER2S trial
Source: Ann Intensive Care. 2018 Sep 17;8:90. doi: 10.1186/s13613-018-0435-1 (PMC6141409; doi:10.1186/s13613-018-0435-1)

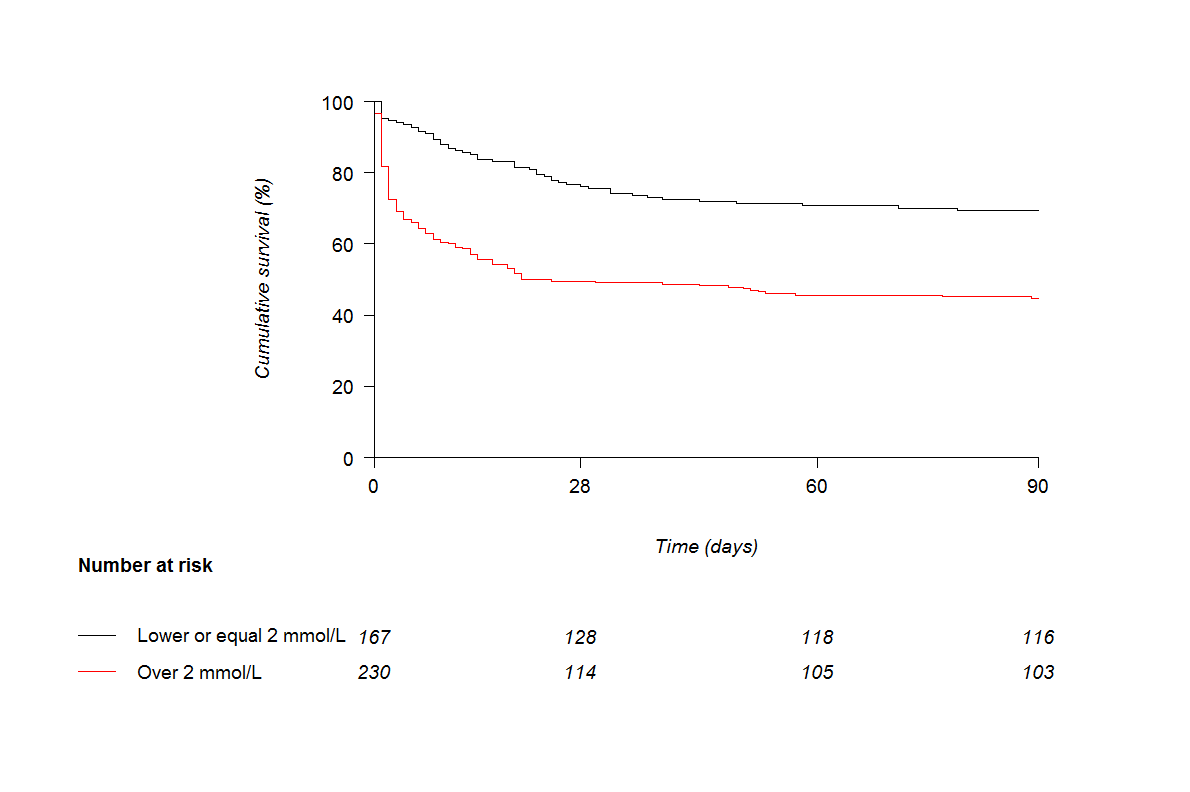

Supplement: Supplementary file 3 — Additional file 3: Figure S1. Kaplan–Meier curves of all patients with hyperlactatemia (lactate > 2 mmol/L) (n = 230) and those with lactates ≤ 2 mmol/L at baseline (n = 167). [file 13613_2018_435_MOESM3_ESM.png]
