# Supplementary material for: Hyperoxia toxicity in septic shock patients according to the Sepsis-3 criteria: a post hoc analysis of the HYPER2S trial
Source: Ann Intensive Care. 2018 Sep 17;8:90. doi: 10.1186/s13613-018-0435-1 (PMC6141409; doi:10.1186/s13613-018-0435-1)
Supplement: Supplementary file 4 — Additional file 4: Table S3. Daily evolution of the SOFA score’s components of patients according to lactate level (≤ 2 or > 2 mmol/L) and normoxia or hyperoxia treatment. [file 13613_2018_435_MOESM4_ESM.docx]

**Additional file 4: Table S3. Daily evolution of the SOFA score’s components of patients according to lactate level (≤ 2 or > 2 mmol/L) and normoxia or hyperoxia treatment.**

For the analysis of SOFA score’s components, Student’s and Man Whitney test were used. d = day, h = hour. For SOFA-Cardiovascular item, no data is presented during the first 24 hours (SOFA-C at 4 for all included patients).

|  | **Lactate ≤ 2 mmol/L (n = 167)** | | | **Lactate >2 mmol/L (n = 230)** | | |
| --- | --- | --- | --- | --- | --- | --- |
|  | **Normoxia (n = 75)** | **Hyperoxia**  **(n = 92)** | **p-value** | **Normoxia**  **(n = 122)** | **Hyperoxia**  **(n = 108)** | **p-value** |
| ***SOFA – Respiration (R)*** | | | | | | |
| SOFA-(R) h0  Mean (SD)  Median (IQR) | 2.7 (0.9)  3 (2-3) | 2.9 (0.9)  3 (2.8-3.2) | 0.187  0.132 | 2.6 (1.1)  3 (2-3) | 2.7 (0.9)  3 (2-3) | 0.304  0.326 |
| SOFA-(R) h24  Mean (SD)  Median (IQR) | 2.5 (0.9)  3 (2-3) | 2.7 (1)  3 (2-4) | 0.173  0.139 | 2.7 (1)  3 (2-3) | 2.8 (1.2)  3 (2-4) | 0.334  0.140 |
| SOFA-(R) h48  Mean (SD)  Median (IQR) | 2.3 (1)  3 (2-3) | 2.8 (1)  3 (2-3) | **0.006**  **0.002** | 2.4 (1)  3 (2-3) | 2.6 (1.1)  3 (2-3) | 0.254  0.225 |
| SOFA-(R) h72  Mean (SD)  Median (IQR) | 2.1 (1)  3 (2-3) | 2.6 (1)  3 (2-3) | **0.002**  **0.002** | 2.2 (1.1)  3 (2-3) | 2.2 (1)  2 (1-3) | 0.937  0.861 |
| SOFA-(R) d4  Mean (SD)  Median (IQR) | 2.2 (0.9)  2 (2-3) | 2.5 (0.9)  3 (2-3) | 0.192  0.124 | 2 (1)  2 (1.8-3) | 2 (1)  2 (1-3) | 0.976  0.982 |
| SOFA-(R) d5  Mean (SD)  Median (IQR) | 2.2 (1)  2 (1.8-3) | 2.3 (1)  2 (2-3) | 0.690  0.619 | 2 (1.2)  2 (1-3) | 2 (1)  2 (1-3) | 0.864  0.962 |
| SOFA-(R) d6  Mean (SD)  Median (IQR) | 2.1 (1)  2 (1-3) | 2.3 (1)  2 (2-3) | 0.362  0.401 | 1.9 (1.3)  2 (1-3) | 2 (1.1)  2 (1-3) | 0.795  0.876 |
| SOFA-(R) d7  Mean (SD)  Median (IQR) | 2.2 (1.1)  2 (1-3) | 2.3 (1.2)  2 (2-3) | 0.939  0.988 | 1.9 (1.2)  2 (1-3) | 1.7 (1)  2 (1-2.5) | 0.317  0.306 |
| ***SOFA – Liver (L)*** | | | | | | |
| SOFA-(L) h0  Mean (SD)  Median (IQR) | 0.4 (0.8)  0 (0-1) | 0.4 (0.8)  0 (0-1) | 0.849  0.871 | 0.6 (0.9)  0 (0-1) | 0.5 (0.8)  0 (0-1) | 0.343  0.360 |
| SOFA-(L) h24  Mean (SD)  Median (IQR) | 0.3 (0.8)  0 (0-0) | 0.3 (0.7)  0 (0-0) | 0.989  0.784 | 0.8 (0.9)  0 (0-1.2) | 0.6 (0.8)  0 (0-1) | 0.297  0.317 |
| SOFA-(L) h48  Mean (SD)  Median (IQR) | 0.4 (0.8)  0 (0-0) | 0.3 (0.7)  0 (0-0) | 0.631  0.745 | 0.7 (0.9)  0 (0-1) | 0.6 (0.8)  0 (0-1) | 0.184  0.175 |
| SOFA-(L) h72  Mean (SD)  Median (IQR) | 0.4 (0.9)  0 (0-0) | 0.3 (0.7)  0 (0-0) | 0.265  0.458 | 0.7 (1)  0 (0-1) | 0.4 (0.7)  0 (0-1) | **0.018**  0.054 |
| SOFA-(L) d4  Mean (SD)  Median (IQR) | 0.4 (0.8)  0 (0-0) | 0.2 (0.6)  0 (0-0) | 0.221  0.165 | 0.6 (1)  0 (0-1) | 0.3 (0.6)  0 (0-0) | **0.029**  0.117 |
| SOFA-(L) d5  Mean (SD)  Median (IQR) | 0.3 (0.7)  0 (0-0) | 0.2 (0.6)  0 (0-0) | 0.262  0.104 | 0.8 (1.1)  0 (0-2) | 0.3 (0.7)  0 (0-0) | **0.006**  **0.011** |
| SOFA-(L) d6  Mean (SD)  Median (IQR) | 0.1 (0.3)  0 (0-0) | 0.2 (0.7)  0 (0-0) | 0.253  0.753 | 0.8 (1.2)  0 (0-1) | 0.4 (0.7)  0 (0-0) | **0.026**  0.054 |
| SOFA-(L) d7  Mean (SD)  Median (IQR) | 0.1 (0.2)  0 (0-0) | 0.2 (0.7)  0 (0-0) | 0.165  0.504 | 0.9 (1.2)  0 (0-2) | 0.3 (0.5)  0 (0-0) | **0.001**  **0.011** |
| ***SOFA – Cardiovascular (C)*** | | | | | | |
| SOFA-(C) h48  Mean (SD)  Median (IQR) | 2.9 (1.5)  4 (1-4) | 3.7 (1)  4 (4-4) | **0.001**  **<0.001** | 3.4 (1.2)  4 (3.8-4) | 3.6 (1.1)  4 (4-4) | 0.268  0.092 |
| SOFA-(C) h72  Mean (SD)  Median (IQR) | 2.1 (1.7)  2 (0-4) | 2.7 (1.7)  4 (1-4) | **0.032**  **0.031** | 2.7 (1.6)  4 (1-4) | 2.7 (1.7)  4 (1-4) | 0.941  0.897 |
| SOFA-(C) d4  Mean (SD)  Median (IQR) | 1.4 (1.7)  1 (0-3.5) | 1.5 (1.7)  1 (0-4) | 0.575  0.515 | 1.4 (1.7)  1 (0-4) | 1.2 (1.5)  1 (0-1.8) | 0.354  0.608 |
| SOFA-(C) d5  Mean (SD)  Median (IQR) | 1.3 (1.6)  1 (0-3) | 1.3 (1.6)  1 (0-1.8) | 0.924  0.994 | 1.5 (1.7)  1 (0-3) | 1.1 (1.5)  1 (0-1) | 0.193  0.374 |
| SOFA-(C) d6  Mean (SD)  Median (IQR) | 1.3 (1.6)  1 (0-3) | 1.2 (1.5)  1 (0-1) | 0.710  0.761 | 1.4 (1.7)  1 (0-3) | 1 (1.4)  0 (0-1) | 0.147  0.272 |
| SOFA-(C) d7  Mean (SD)  Median (IQR) | 1.1 (1.5)  1 (0-1) | 1 (1.5)  1 (0-1) | 0.705  0.670 | 1.4 (1.7)  1 (0-3.8) | 0.9 (1.3)  0.5 (0-1) | 0.070  0.231 |
| ***SOFA – Central Nervous System (CNS)*** | | | | | | |
| SOFA-(CNS) h0  Mean (SD)  Median (IQR) | 0.7 (1.1)  0 (0-1) | 0.7 (1.2)  0 (0-1) | 0.831  0.953 | 0.9 (1.3)  0 (0-1) | 0.7 (1.2)  0 (0-1) | 0.475  0.426 |
| SOFA-(CNS) h24  Mean (SD)  Median (IQR) | 0.7 (1.1)  0 (0-1) | 0.7 (1.2)  0 (0-1) | 0.819  0.886 | 0.8 (1.2)  0 (0-1) | 0.8 (1.2)  0 (0-1) | 0.993  0.830 |
| SOFA-(CNS) h48  Mean (SD)  Median (IQR) | 0.5 (0.9)  0 (0-1) | 0.7 (1.2)  0 (0-1) | 0.422  0.864 | 0.8 (1.2)  0 (0-1) | 0.8 (1.2)  0 (0-1.5) | 0.895  0.902 |
| SOFA-(CNS) h72  Mean (SD)  Median (IQR) | 0.5 (0.9)  0 (0-1) | 0.7 (1.1)  0 (0-1) | 0.261  0.549 | 0.8 (1.3)  0 (0-1.8) | 0.8 (1.2)  0 (0-2) | 0.943  0.992 |
| SOFA-(CNS) d4  Mean (SD)  Median (IQR) | 0.4 (0.9)  0 (0-0) | 0.6 (1.1)  0 (0-1) | 0.314  0.380 | 0.8 (1.2)  0 (0-1) | 0.6 (1.1)  0 (0-1) | 0.458  0.402 |
| SOFA-(CNS) d5  Mean (SD)  Median (IQR) | 0.4 (1)  0 (0-0) | 0.6 (1.1)  0 (0-1) | 0.498  0.455 | 0.8 (1.3)  0 (0-1.2) | 0.6 (1.1)  0 (0-1) | 0.406  0.386 |
| SOFA-(CNS) d6  Mean (SD)  Median (IQR) | 0.4 (1)  0 (0-0) | 0.6 (1.1)  0 (0-1) | 0.286  0.384 | 0.8 (1.2)  0 (0-2) | 0.7 (1.2)  0 (0-1) | 0.463  0.363 |
| SOFA-(CNS) d7  Mean (SD)  Median (IQR) | 0.4 (1)  0 (0-0) | 0.6 (1.2)  0 (0-1) | 0.194  0.201 | 0.9 (1.2)  0 (0-2) | 0.6 (1.1)  0 (0-1) | 0.299  0.248 |
| ***SOFA – Renal (Ren)*** | | | | | | |
| SOFA-(Ren) h0  Mean (SD)  Median (IQR) | 1 (1.2)  1 (0-2) | 1.2 (1.4)  1 (0-2) | 0.351  0.514 | 1.8 (1.6)  1.5 (0-3.2) | 1.7 (1.5)  1 (0-3) | 0.740  0.793 |
| SOFA-(Ren) h24  Mean (SD)  Median (IQR) | 1.1 (1.4)  1 (0-2) | 1.5 (1.6)  1 (0-3) | 0.062  0.068 | 2.5 (1.7)  3 (1-4) | 2.3 (1.6)  3 (1-4) | 0.531  0.388 |
| SOFA-(Ren) h48  Mean (SD)  Median (IQR) | 0.8 (1.3)  0 (0-1) | 1.5 (1.7)  1 (0-4) | **0.007**  **0.019** | 2.1 (1.8)  2 (0-4) | 2.1 (1.7)  2 (0-4) | 0.801  0.845 |
| SOFA-(Ren) h72  Mean (SD)  Median (IQR) | 0.8 (1.3)  0 (0-1) | 1.3 (1.7)  0 (0-3) | **0.045**  0.060 | 1.9 (1.8)  2 (0-4) | 1.8 (1.8)  1 (0-4) | 0.621  0.646 |
| SOFA-(Ren) d4  Mean (SD)  Median (IQR) | 0.9 (1.4)  0 (0-1.5) | 1.2 (1.6)  0 (0-2) | 0.234  0.233 | 1.5 (1.7)  1 (0-3) | 1.3 (1.7)  1 (0-3) | 0.513  0.463 |
| SOFA-(Ren) d5  Mean (SD)  Median (IQR) | 0.8 (1.4)  0 (0-1) | 1.1 (1.6)  0 (0-2) | 0.201  0.169 | 1.6 (1.7)  1 (0-4) | 1.3 (1.7)  0 (0-2.8) | 0.305  0.255 |
| SOFA-(Ren) d6  Mean (SD)  Median (IQR) | 0.8 (1.3)  0 (0-1) | 1 (1.5)  0 (0-1) | 0.401  0.457 | 1.6 (1.7)  1 (0-4) | 1.4 (1.7)  0 (0-3.5) | 0.512  0.400 |
| SOFA-(Ren) d7  Mean (SD)  Median (IQR) | 0.7 (1.2)  0 (0-1) | 1 (1.5)  0 (0-1) | 0.254  0.389 | 1.8 (1.8)  1 (0-4) | 1.4 (1.7)  0 (0-3.5) | 0.220  0.181 |
| ***SOFA – Coagulation (Co)*** | | | | | | |
| SOFA-(Co) h0  Mean (SD)  Median (IQR) | 0.6 (1.2)  0 (0-1) | 0.4 (0.9)  0 (0-0) | 0.126  0.136 | 0.8 (1.1)  0 (0-1) | 0.8 (1.2)  0 (0-1) | 0.886  0.775 |
| SOFA-(Co) h24  Mean (SD)  Median (IQR) | 0.7 (1.1)  0 (0-1) | 0.5 (1)  0 (0-1) | 0.248  0.150 | 1.2 (1.2)  1 (0-2) | 1 (1.2)  0 (0-2) | 0.310  0.236 |
| SOFA-(Co) h48  Mean (SD)  Median (IQR) | 0.8 (1.2)  0 (0-1) | 0.8 (1.1)  0 (0-1) | 0.825  0.877 | 1.5 (1.4)  1 (0-2) | 1.4 (1.4)  1 (0-2) | 0.569  0.534 |
| SOFA-(Co) h72  Mean (SD)  Median (IQR) | 0.7 (1.1)  0 (0-1) | 0.8 (1.2)  0 (0-1) | 0.667  0.715 | 1.6 (1.3)  2 (0-2) | 1.4 (1.3)  1 (0-3) | 0.423  0.430 |
| SOFA-(Co) d4  Mean (SD)  Median (IQR) | 0.7 (1.1)  0 (0-2) | 0.8 (1.3)  0 (0-1) | 0.699  0.901 | 1.4 (1.4)  1 (0-2) | 1.2 (1.3)  1 (0-2) | 0.463  0.564 |
| SOFA-(Co) d5  Mean (SD)  Median (IQR) | 0.7 (1)  0 (0-1) | 0.8 (1.2)  0 (0-1) | 0.564  0.789 | 1.2 (1.3)  1 (0-2) | 1.1 (1.3)  1 (0-2) | 0.697  0.732 |
| SOFA-(Co) d6  Mean (SD)  Median (IQR) | 0.7 (1.1)  0 (0-1) | 0.6 (1.1)  0 (0-0.8) | 0.633  0.482 | 1 (1.2)  0 (0-2) | 1 (1.2)  1 (0-2) | 0.713  0.601 |
| SOFA-(Co) d7  Mean (SD)  Median (IQR) | 0.6 (1.1)  0 (0-1) | 0.5 (1.1)  0 (0-0) | 0.594  0.348 | 1 (1.3)  0 (0-2) | 0.9 (1.1)  0 (0-2) | 0.790  0.950 |
