# Supplementary material for: Hyperoxia toxicity in septic shock patients according to the Sepsis-3 criteria: a post hoc analysis of the HYPER2S trial
Source: Ann Intensive Care. 2018 Sep 17;8:90. doi: 10.1186/s13613-018-0435-1 (PMC6141409; doi:10.1186/s13613-018-0435-1)
Supplement: Supplementary file 5 — Additional file 5: Table S4. Infectious events during the ICU stay of patients according to lactate level (≤ 2 or > 2 mmol/L) and normoxia or hyperoxia treatment. [file 13613_2018_435_MOESM5_ESM.docx]

**Additional file 5: Table S4. Infectious events during the ICU stay of patients according to lactate level (≤ 2 or > 2 mmol/L) and normoxia or hyperoxia treatment.**

The Chi-square or Fisher-Test was used for qualitative data. The quantitative data were compared by Mann-Witney-Test for median comparisons. p-values are presented for the comparison between “normoxia” and “hyperoxia” treatment in the lactate ≤ 2 mmol/L and lactate>2mmol/L groups respectively. IQR= interquartile range.

|  | **Lactate ≤ 2 mmol/L (n = 167)** | | | **Lactate >2 mmol/L (n = 230)** | | |
| --- | --- | --- | --- | --- | --- | --- |
|  | **Normoxia**  **(n = 75)** | **Hyperoxia**  **(n = 92)** | **p-value** | **Normoxia**  **(n = 122)** | **Hyperoxia**  **(n = 108)** | **p-value** |
| Patients with ≥ 1 nosocomial infection, n (%) | 14 (18.7%) | 26 (28.3%) | 0.149 | 19 (15.6%) | 10 (9.3%) | 0.150 |
| Delay between randomization and infection (days),  Mean (IQR) | 8 (6-14) | 13 (7-17) | 0.245 | 7 (5-16) | 12 (7-22) | 0.143 |
| Infection site |  |  |  |  |  |  |
| Lung, n (%) | 11 (14.7%) | 22 (23.9%) | 0.136 | 18 (14.8%) | 4 (3.7%) | **0.009** |
| Bacteriemia, n (%) | 6 (8%) | 3 (3.3%) | 0.177 | 5 (4.1%) | 3 (2.8%) | 0.585 |
| Urinary, n (%) | 0 (0%) | 4 (4.3%) | 0.128 | 1 (0.8%) | 3 (2.8%) | 0.344 |
| Surgical site infection, n (%) | 0 (0%) | 2 (2.2%) | 0.502 | 0 (0%) | 1 (0.9%) | 0.450 |
| Catheter related, n (%) | 1 (1.3%) | 2 (2.2%) | 1 | 3 (2.5%) | 1 (0.9%) | 0.625 |
| Infection responsible for new septic shock, n (%) | 2 (2.7%) | 4 (4.3%) | 0.692 | 5 (4.1%) | 3 (2.8%) | 0.853 |
