# Supplementary material for: Hyperoxia toxicity in septic shock patients according to the Sepsis-3 criteria: a post hoc analysis of the HYPER2S trial
Source: Ann Intensive Care. 2018 Sep 17;8:90. doi: 10.1186/s13613-018-0435-1 (PMC6141409; doi:10.1186/s13613-018-0435-1)
Supplement: Supplementary file 6 — Additional file 6: Table S5. Data analysis by using a Cox regression model with survival data censored at 28 days and then censored at 90 days in succession, excluding cirrhotic patients. HR hazard ratio, CI confidence interval, F female, M male. [file 13613_2018_435_MOESM6_ESM.docx]

| Univariate Cox model (universal analysis) | | | | | | | | |
| --- | --- | --- | --- | --- | --- | --- | --- | --- |
|  | Inclusion d28 | | | | Inclusion d90 | | | |
|  | HR | 95% CI | | p-value | HR | 95%CI | | p-value |
| Sex (F vs M) | 1.37 | 0.98 | 1.90 | 0.065 | 1.53 | 1.06 | 2.21 | **0.022** |
| Weight (per 10 kg increase) | 0.97 | 0.85 | 1.10 | 0.635 | 1.01 | 0.90 | 1.15 | 0.800 |
| pH (per 0.1 increase) | 0.97 | 0.95 | 0.98 | **<0.001** | 0.97 | 0.96 | 0.99 | **<0.001** |
| Hyperoxia vs normoxia | 1.54 | 1.05 | 2.27 | **0.027** | 1.35 | 0.94 | 1.95 | 0.104 |
| SAPS 2 | 1.05 | 1.04 | 1.06 | **<0.001** | 1.05 | 1.04 | 1.06 | **<0.001** |
| Mac Cabe | 1.25 | 0.95 | 1.65 | 0.112 | 1.26 | 0.97 | 1.64 | 0.088 |
| PaO_2_/FiO_2_ | 1 | 0.99 | 1.00 | 0.967 | 1 | 0.99 | 1.00 | 0.520 |
| Autoimmune disease | 1.19 | 0.76 | 1.86 | 0.459 | 1.29 | 0.84 | 1.96 | 0.241 |
| Multivariate Cox model | | | | | | | | |
|  | Inclusion d28 | | | | Inclusion d90 | | | |
|  | HR | 95%CI | | p-value | HR | 95% CI | | p-value |
| Sex (F vs M) | 1.54 | 1.03 | 2.30 | **0.034** | 1.48 | 1.01 | 2.17 | **0.0425** |
| pH (per 0.1 increase) | 0.97 | 0.95 | 0.99 | **<0.001** | 0.97 | 0.95 | 0.99 | **<0.001** |
| Hyperoxia vs normoxia | 1.95 | 1.29 | 2.95 | **0.002** | 1.69 | 1.15 | 2.50 | **0.008** |
| SAPS 2 | 1.05 | 1.04 | 1.07 | **<0.001** | 1.05 | 1.04 | 1.07 | **<0.001** |
| Mac Cabe | 1.03 | 0.78 | 1.36 | 0.853 | 1.02 | 0.78 | 1.34 | 0.640 |

**Additional file 6: Table S5. Data analysis by using a Cox regression model with survival data censored at 28 days and then censored at 90 days in succession, excluding cirrhotic patients.** HR=hazard ratio. CI=confidence interval. F=female. M=male.
